# Examination on the Occurrence of Coinfections in Diagnostic Transmittals in Cases of Stillbirth, Mummification, Embryonic Death, and Infertility (SMEDI) Syndrome in Germany

**DOI:** 10.3390/microorganisms11071675

**Published:** 2023-06-27

**Authors:** Matthias Eddicks, Julia Gründl, Annika Seifert, Lina Eddicks, Sven Reese, Robert Tabeling, Hanny Swam, Katrin Strutzberg-Minder, Mathias Ritzmann, Robert Fux

**Affiliations:** 1 Clinic for Swine at the Centre for Clinical Veterinary Medicine, Ludwig-Maximilians-Universität München, 85764 Oberschleissheim, Germany; julia.gruendl@med.vetmed.uni-muenchen.de (J.G.); annika.seifert@med.vetmed.uni-muenchen.de (A.S.); m.ritzmann@lmu.de (M.R.); 2 Institute of Veterinary Pathology at the Centre for Clinical Veterinary Medicine, Ludwig-Maximilians-Universität München, 80539 München, Germany; lina.eddicks@lmu.de; 3 Institute for Anatomy, Histology and Embryology, Department of Veterinary Sciences, Ludwig-Maximilians-Universität München, 80539 München, Germany; sven.reese@lmu.de; 4 MSD Animal Health, Intervet Deutschland GmbH, 85716 Unterschleissheim, Germany; robert.tabeling@msd.de; 5 Intervet International B.V., 5831 AK Boxmeer, The Netherlands; hanny.swam@merck.com; 6 IVD Innovative Veterinary Diagnostics (IVD GmbH), DVG-Consiliary Laboratory for Leptospira spp., 30926 Seelze, Germany; strutzberg@ivd-gmbh.de; 7 Division of Virology, Department of Veterinary Sciences, Ludwig-Maximilians-Universität München, 85764 Oberschleissheim, Germany; robert.fux@lmu.de

**Keywords:** porcine parvovirus 1, porcine circovirus 2, *Leptospira* spp., porcine circovirus 3, reproductive disease, swine, fetus, mummification, maceration

## Abstract

The stillbirth, mummification, embryonic death, and infertility (SMEDI) syndrome is most commonly associated with porcine parvovirus 1 (PPV1) infections. Little is known about the occurrence of coinfections with SMEDI-associated pathogens and the associations among these pathogens. In our study, we included 40 SMEDI-affected litters from 18 different farms. In total, 158 out of 358 available fetuses from diagnostic transmittals were selected by systematic random sampling and examined for PCV2, PCV3, PPV1, and *Leptospira* spp. by q-PCR. Results from diagnostic materials showed the following results: in eleven farms, PCV2 was present; in nine farms, PPV1 was present; in five farms, PCV3 was present; and in two farms, *Leptospira* spp. was present. The detection of *Leptospira* spp. was significantly associated with a PCV2 coinfection (OR: 26.3; *p* < 0.001). PCV3 positivity resulted in a reduced probability of detecting PCV2 in the corresponding fetus (OR: 0.078; *p* = 0.008). Fetal maceration was associated with *Leptospira* spp. detection (OR: 8.6; *p* = 0.003), whereas mummification (*p* = 0.047), reduced crown-rump length (*p* < 0.001), and bodyweight (*p* = 0.001) of fetuses were significantly associated with PPV1 and PCV2 coinfection and thus, presumably, a shorter time to death after infection, indicating an enhanced negative effect on the development of fetuses with PCV2 + PPV1 coinfection.

## 1. Introduction

Reproductive disorders in sows include a wide variety of clinical appearances. Among others, the SMEDI (stillbirth, mummification, embryonic death, and infertility) syndrome displays a very characteristic presentation of a disturbed gestation in sows. The clinical picture of the SMEDI syndrome depends on the time of infection of susceptible dams, accompanied by the immune status of the corresponding fetuses. Infection of the embryos of susceptible sows leads to resorption of the embryos, with the consequence of a reduced litter size. The typical appearance of SMEDI-affected litters is assumed to depend on a successive horizontal transmission of the involved pathogen within the uterus that goes along with intrauterine fetal death. Affected litters may include mummified, macerated, or stillborn fetuses with an ascending crown-rump length, depending on the age at the time of infection. Fetuses older than 70 days of gestation are assumed to be immunocompetent [1], and infections at that stage of pregnancy might lead to weak or live-born piglets. The major pathogen that is associated with the occurrence of SMEDI in sow farms is the ungulate protoparvovirus 1, commonly called porcine parvovirus 1 (PPV1) [2,3]. However, porcine circovirus 2 (PCV2) [4,5,6,7] or Leptospira spp. [8,9] are also regularly reported or taken into consideration as a differential diagnosis in such cases. However, prevalence data or detection rates of the before-mentioned pathogens in well-defined SMEDI cases are rare. In general, it is assumed that high vaccination rates and improved health management may have led to a low prevalence of PPV1 in such cases [10]. However, Belgium and the Netherlands have seen an increase in PPV1-associated SMEDI cases in recent years, probably due to new emerging PPV1 variants [3]. Concerning PCV2, SMEDI cases are regularly reported in the field [5,11,12,13] or after experimental infection [6]. The overall prevalence of PCV2-associated reproductive disorders, also known as PCV2-reproductive disease [14], seems to be low and limited to young sows, appearing mostly in herds with high numbers of gilts [1,11]. An exact prevalence of leptospiral infections in cases of reproductive disorders, particularly in cases of SMEDI, is not available. Epidemiological information based on MAT (microscopic agglutination test) results of diagnostic transmittals indicates high seroprevalences in Germany [15] but does not reflect the etiological role of Leptospira spp. in cases of reproductive disorders, or more concretely, in the case of SMEDI. Another agent associated with reproductive disorders in sows, including stillbirth and mummification, is the porcine circovirus 3 (PCV3) [16,17,18]. Although the pathogenicity of PCV3 is still under discussion, PCV3-associated diseases are already defined. In addition to a so-called PCV3-systemic disease, a PCV3-reproductive disease was also suggested [19]. 

In terms of coinfections, it is well known that double infection with PPV1 and PCV2 leads to an increase in PCV2-associated clinical and pathomorphological changes and higher PCV2-DNA loads in the tissue of young pigs under controlled conditions [20,21,22]. In terms of reproductive parameters, a possible effect in sows was demonstrated in an experimental setting [6], when SMEDI-like appearances of litters from PCV2-infected non-PPV1 + erysipelas-vaccinated sows were observed, whereas litters from PCV2-infected sows vaccinated against PPV1 and erysipelas did not show noticeable problems. However, less is known about the occurrence of coinfections with different pathogens in the case of SMEDI and its possible effects on the affected litters. In the present study, we examined SMEDI-litters that were transferred for diagnostic purposes from German piggeries for the presence of PPV1, PCV2, PCV3, and Leptospira spp. by q-PCR. All litters were photo-documented, and crown-rump length and bodyweight were noted. Furthermore, questionnaire-based farm-specific information concerning vaccinations, parity of the dams, and litter size was evaluated. The gained data was set in correlation, and pathogen associations were determined.

## 2. Material and Methods

### 2.1. Sample Material

For the present study, diagnostic transmittals in cases of SMEDI-associated reproductive disorders were included. The referring veterinarians were asked to send in the whole SMEDI-affected litter (only dead fetuses) for the diagnostic examinations. All litters were deep-frozen when they reached our facilities.

**Figure 1:**
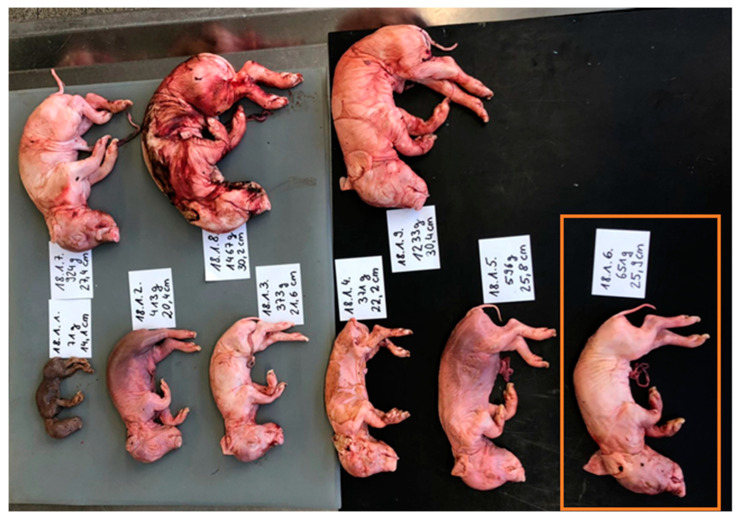
Example of the photo documentation of a SMEDI litter (farm 18). The fetuses of each litter were sorted by size. Each fetus received an individual lab number (farm.litter.fetus -> e.g., orange box: 18.1.6), was weighted (g), measured (cm), and assigned to a phenotypical type of appearance (mummified, stillborn-macerated, stillborn-fresh, and weak-born (positive lung floating test).

For each transmittal, information on the parity of the corresponding sow, total litter size (including those not sent in live-born animals), and vaccination strategies were noted. All litters were photo documented, and from all fetuses, the crown-rump length, bodyweight, phenotypical appearance (mummy, stillborn-macerated, and stillborn-fresh), and sex were documented. An exemplary depiction is presented in Figure 1. Detailed information on the herd size, vaccination against PPV1, PCV2, and *Leptospira* spp. of the sows, as well as sampled fetuses and the farm, is available in Supplementary File S1.

### 2.2. Sampling

Within our routine diagnostics, we sampled four fetuses per litter, as recommended elsewhere for the diagnosis of abortion and reproductive diseases [23]. To ensure all age groups/sizes were included in our examinations of each litter, we selected the fetuses by systematic random sampling with respect to the litter size. To calculate the sample interval for each litter, we divided the number of available fetuses by four. The calculated number was rounded up and depicted the sample interval beginning (e.g., number of available fetuses is nine: 9/4 = 2.25; every second fetus will be examined). To further consider the different phenotypes, we shifted the starting point for each litter, starting at one up to four and then starting at one again. If only four fetuses were available, all of the fetuses from this litter were chosen. 

### 2.3. Sample Collection

After thawing at room temperature, the fetuses were opened in a supine position. A lung floating test was carried out to examine whether the fetus was stillborn or weak-born. From each fetus, two tissue pools were created. One for the examination for PCV2, PCV3, and PPV1 DNA (myocardium, lung, thymus, and spleen) and one for the detection of *Leptospira* ssp. DNA (meconium, stomach contents, liver, and kidney). The pooled samples received the same number as depicted in Figure 1. To avoid cross contamination of tissues, the set of instruments for the sample collection was cleaned, dipped in alcohol (96%), and flame-treated after each sampling.

### 2.4. Molecular Biological Examinations

#### 2.4.1. Viral Pathogens

DNA from homogenized organ pools was isolated using commercially available kits (e.g., QIAamp DNA Mini Kit, Qiagen, MagNA Pure DNA, and Viral NA small volume kit, Roche) according to the manufacturer’s instructions. Published q-PCR assays were used for the specific detection of PPV1 [24] and PCV2 [25] DNA, respectively. For the detection of PCV3, a commercially available assay (EXOone PCV3, exopol) was used. For the viral pathogens, a Cq-value of ≤35 was rated as positive.

#### 2.4.2. *Leptospira* spp.

The Mag-MAX™ Pathogen RNA/DNA kit, Applied Biosystems, was used for DNA isolation according to the manufacturer’s protocol. The Lipl32 multiplex real-time PCR assay described by Ferreira et al. [26] was used for the detection of pathogenic *Leptospira* spp. A sample was rated as positive when the Cq-value of the PCR was <40.

### 2.5. Statistical Analysis

Collected data, including the diagnostic results, were documented as metric scale (e.g., Cq-values) and encoded in binary data (e.g., PCR positive/negative) in Excel. Statistical calculations were performed with the software IBM SPSS Statistics version 28.0.1.0 for Microsoft^®^ Windows. The prevalence was specified as absolute and relative frequency in percent with a 95% confidence interval. The significance level was *p* < 0.05. Binary data were first checked for associations between independent and dependent variables by a univariate analysis (Chi^2^). Subsequently, we conducted a multivariable analysis (Generalized linear model, GLM) that included all significantly associated independent factors. Generalized estimating equations (GEE), a special form of the GLM, were used to account for the clustering of the data (herds and litters). A binomial logistic probability distribution was chosen as the model type for binary dependent variables. For all results, the odds ratio and the corresponding 95% confidence interval were calculated. As dependent variables for this analysis, we chose the qualitative PCR results for each pathogen. Independent variables included: Parity (gilt/old sow) and vaccination of the sows (PCV2, PPV1, Erysipelas, *Leptospira* spp., PRRSV) and the outcome for each pathogen of the corresponding litters.

Metric data were tested for normal distribution by the Kolmogorov-Smirnov test. In cases where the data were not normally distributed, non-parametric tests for independent data were used (two independent samples: Mann-Whitney-U-Test; more than two independent samples: Kruskal-Wallis-Test with Bonferroni adjustment of the *p*-value). Concretely, we tested for associations between all coinfection combinations as independent variables and the quantitative PCR results (Cq-value) of the corresponding pathogen as a dependent variable. A bivariate non-parametric correlation according to Spearman was conducted to evaluate possible correlations between the quantitative outcome of the PCR results (Cq-value) for each pathogen, the crown-rump length, and the bodyweight of the fetuses. To estimate the effect of coinfections on the phenotypical appearance, SSL, and crown-rump length Generalized estimating equations (GEE), a special form of the GLM, were used to account for the clustering of the data (herds and litters). Gamma was chosen as the model type for metric-dependent variables.

## 3. Results

### 3.1. Study Population

**Figure 2:**
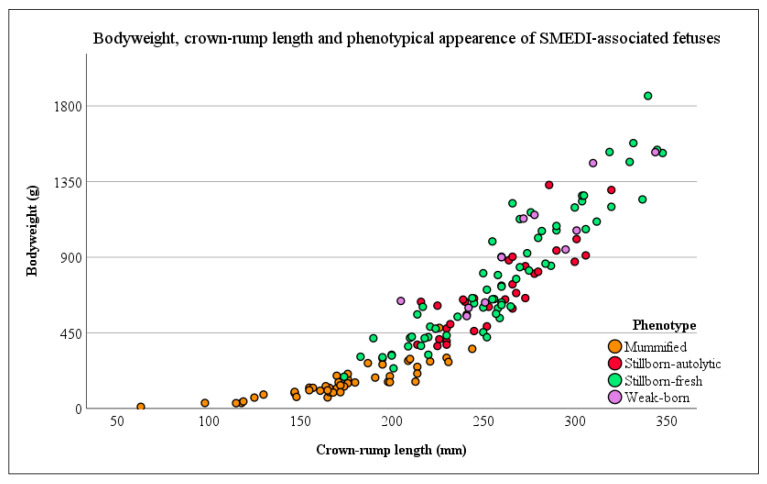
Crown-rump length and bodyweight of the sampled fetuses with respect to the phenotypical classification (different colors). Each dot represents a single fetus.

Out of a pool of 358 fetuses with a crown-rump length of 232.35 mm (±54.22 mm), 158 fetuses with a mean crown-rump length of 233.98 mm (±55.07 mm) were enrolled in this examination. The distribution of these fetuses on different phenotypes is shown for all (n = 358) and for the study population (n = 158) in Table 1. A detailed view of the crown-rump length, bodyweight, and phenotypical appearance of single fetuses is provided in Figure 2.

### 3.2. Molecular Biological Examinations

#### 3.2.1. Detection of Single Pathogens

In 17 of 18 farms, one or more pathogens were detected in at least one fetus within the sample population. In detail, the following was found in SMEDI litters: on eleven farms, PCV2 DNA was detected; on nine farms, PPV DNA was detected; on five farms, PCV3 DNA was detected; and on two farms, *Leptospira* spp. DNA was detected. Detailed information on the detection rate of each single pathogen, including farms, litters, and single fetuses, is available in Table 2. The PCR results for each single farm are available in the Supplemental Material (S2).

To evaluate the probability of detection for each pathogen (dependent variable) in association with available farm- or fetus-specific factors (independent factors: *fetus mummified*, *fetus stillborn-macerated*, *fetus PPV1-positive*, *fetus PCV2-positive*, *fetus PCV3-positive*, *fetus Leptospira* spp.*-positive*; *PCV2 sow vaccination*, *Leptospira* spp. *sow vaccination*; *PPV1 sow vaccination*) a multivariable analysis including all significantly associated factors from a preceding univariate analysis was conducted. This examination revealed a significant increase in the probability of detection for *Leptospira* spp. in cases of the phenotypical appearance *stillborn-macerated* and in cases of a coinfection with PCV2. Whereas litters from gilts had a significantly decreased chance of being *Leptospira* spp.-positive. More detailed results of this analysis are depicted in Table 3. 

#### 3.2.2. Detection of (Co)infections in Terms of SMEDI

In total, nine different statuseses of infection could be detected in individual fetuses. A detailed presentation of the detection of the different pathogens in farm litter and fetuses is presented in Table 4.

The different statuses of (co)infections had no significant influence on the viral load of the single pathogens in the corresponding fetuses. Table 5 provides the mean Cq-values for each pathogen in the case of different (co)infections. Detailed information on the Cq-values for each pathogen on each single farm is available in Supplementary File S3.

**Figure 3:**
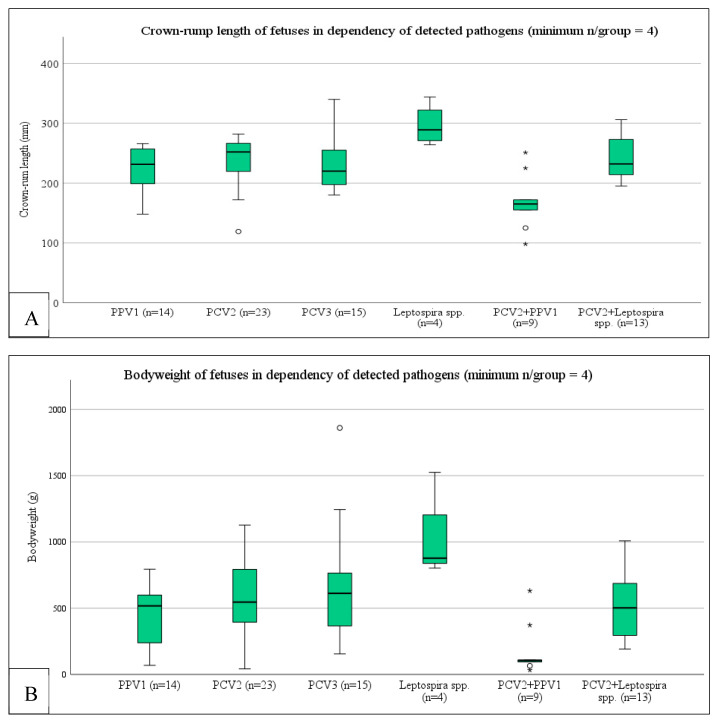
Boxplot of crown-rump length (**A**) and bodyweight (**B**) dependency of the infectious status of SMEDI-associated fetuses. The circle (°) indicates an outlier in the data. The asterisk (*) indicates that there is an extreme outlier in the data.

A bivariate Spearman’s correlation between the variables *Cq-value PPV1*, *Cq-value PCV2*, *Cq-value PCV3*, and *Cq-value Leptospira* spp. and the outcome variables *crown-rump length* and *bodyweight* revealed a significant correlation between the Cq-value of PPV1 in positive tissue pools and the crown-rump length (mm) (*p* = 0.002; r_s_: 0.602) and bodyweight (g) of the fetuses (*p* = 0.001; r_s_: 0.629). To consider the effect of coinfections on the crown-rump length and bodyweight and the phenotypical appearance of the fetuses, we subsequently conducted generalized estimating equations (GEE) with crown-rump length, bodyweight, or phenotypical appearance as outcome (dependent) variables and all possible coinfections as fixed factors. This testing revealed that the crown-rump length (*p* = 0.001) and the bodyweight (*p* = 0.001) of the fetuses were significantly associated with the coinfection of PCV2 and PPV1 as well as with leptospiral infection (Figure 3). The occurrence of mummies in SMEDI-litters was associated with a PPV1 + PCV2 coinfection (*p* = 0.047), whereas autolytic fetuses were associated with leptospiral infection (*p* < 0.001) (either alone or with a PCV2 coinfection).

## 4. Discussion

The present field study was conducted to provide insight into the current situation regarding the occurrence of (co)infections in cases of SMEDI syndrome in Germany. Furthermore, we evaluated possible associations between pathogens and the molecular biological examination results in terms of coinfections. Whereas PPV1 [2] and, meanwhile, PCV2 [5,11] are clearly associated with the SMEDI syndrome, there are only a few reports on Leptospira spp. infections in association with SMEDI. However, leptospiral infections are regularly mentioned as a relevant differential in such cases [8,9]. A clear association between SMEDI and PCV3 has not been reported yet. However, based on the large number of reports concerning reproductive disorders [16,17,27] and a newly defined disease proposal (PCV3-reproductive disease) [19], we decided to include PCV3 in our examinations in a pilot study manner.

In 94.4% of the farms and 77.5% of the litters, we were able to detect either PPV1, PCV2, PCV3, or Leptospira spp. DNA in tissue samples from SMEDI-associated fetuses, alone or in different combinations. This result points out that the number of collected samples was sufficient to detect the desired pathogens in our sample population. In 50.0% of farms, one pathogen was present; in 38.8% of farms, two pathogens were present; and in 11.1% of all farms, three pathogens were present in at least one of the fetuses at the same time. From this perspective, coinfections with two or more of the mentioned pathogens seem to appear regularly on SMEDI-affected farms. Concerning the parity of the sows from SMEDI-affected litters, five sows were gilts, and 35 sows were defined as multipara. Statistically, Leptospira spp. were less often detected in litter from gilts compared to multipara sows. This finding could possibly be explained by the high susceptibility of newly introduced naïve gilts in the main herd to an endemic leptospiral infection. Relating to PCV2, no significant association with parity was obvious. These results were surprising, as gilts are normally seen as a risk group for infectious diseases. Particularly in the case of PCV2-RD, gilts or young sows are associated with clinical outbreaks [11,28,29]. Possibly, the routine vaccination against PCV2 within the quarantine led to less susceptible replacement gilts after their introduction into the main herd. The change in epidemiology of PCV2-infection under the different PCV2-vaccination strategies is also extensively discussed in a review by Segales and Sibila [30]. However, vaccination of gilts might reduce the infectious pressure and susceptibility to clinical outbreaks in this age group. Nevertheless, as we were working with diagnostic transmittals from the field, we could not exclude a sample bias concerning parity.

PCV2 was the most often detected pathogen and was present in 61.1% of all SMEDI-affected farms and in 50% of all corresponding litters. An association between PCV2 and SMEDI is nowadays an accepted finding and seems to be linked to young sows, especially gilts [1,5,11,12,30]. A positive effect of PCV2 sow vaccination on PCV2-associated reproductive failure was recently reported from Norway [11]. Accordingly, we recognized sow vaccination against PCV2 as a protective factor concerning the detection of PCV2 DNA in SMEDI fetuses. However, diaplacental colonization or infection of fetuses also occurs without clinical signs and might display a relevant route of the spread of the virus [29,31]. A greater diagnostic value concerning PCV2 infection displays the correlation between the viral load in serum or tissue samples and the degree of the clinical outcome. For growing-to-finish pigs [32,33] as well as for fetuses in terms of PCV2-RD [14,34], this correlation finds broad acceptance. To define an etiological diagnosis concerning PCV2 in terms of porcine circovirus diseases (PCVD), pathomorphological examinations are usually required [14,35]. However, a recent publication pleaded to only focus on high viral loads, as histopathological lesions in cases of PCV2-RD in the presence of high viral loads might not always be present [34]. As we did not include pathomorphological examinations due to freezing artifacts, our findings are based only on the detection of pathogen-specific DNA in the fetuses and the semi-quantitative evaluation of the PCR results based on the Cq-values in the clinically affected fetuses from SMEDI-litters. Based on the assumption that high viral loads are associated with the clinical outcome in cases of PCVD, on five of eleven PCV2-positive farms, Cq-values below 30 were present, of which three were only positive for PCV2 and the others had a coinfection with PPV1 or Leptospira spp., respectively. In addition to its involvement as an etiological agent in cases of SMEDI, PCV2 is also known as a relevant factor concerning the outcome of other diseases or clinical syndromes, such as the porcine respiratory disease complex (PRDC) [36,37,38]. In relation to this, we detected a significant association between the detection rate of Leptospira spp. in SMEDI-fetuses and a coinfection with PCV2. Based on our results, the chance to detect Leptospira spp. DNA was about 26 times higher in cases of PCV2 coinfection in our study. This observation is markedly different from other surveys on the topic of the detection of pathogens in stillborn or mummified fetuses, where no clear associations between pathogens were detected [39,40,41]. These differences might be explained by the sampled population and the number of samples per affected litter that were examined in the before-mentioned studies. Within these examinations, no or limited information on the overall appearance of the litter is available. Moreover, sporadic fetal death followed by mummification occurs often in litters without a clear clinical appearance and might have multiple non-infectious reasons [39]. In contrast to this, we included only well-characterized SMEDI-associated litters and evenly distributed our sample collection within the litters by systematic random sampling based on crown-rump length. The relatively high PCV2 Cq-values in cases of leptospiral and PCV2 coinfection point out that PCV2 might not have been the driving force for the clinical outcome (14, 29). However, it remains within the realm of possibility that PCV2 facilitates a leptospiral infection in cases of SMEDI. 

In contrast to an increased probability of detecting Leptospira spp. in association with PCV2, we observed a reduced probability of detection concerning PCV2 in our study when PCV3 was present in a fetus. This finding indicates a possible inhibitory effect of PCV3 on the pathogenesis of diaplacental PCV2 infections in fetuses. Maybe limited resources for replication in combination with the time of infection might play a role in this observation, as cardiomyocytes are the main targets for viral replication for both viruses in fetal development [27,42]. However, in earlier examinations of the occurrence of PCV3 and PCV2 in suckling piglets [31], we recognized no associations between both pathogens. 

PPV1 was detected in 27.5% of the SMEDI-litters originating from 50% of all included farms. The relevance of PPV1 concerning the SMEDI syndrome is unequivocal. Several studies, including controlled [43,44] and field studies [45,46], on this topic have been published and reviewed [2,8]. Although the sole detection of PPV1 in clinically affected fetuses displays a reliable procedure for diagnostics [8], we assume that higher viral loads might indicate more clinical relevance. Thus, in three of the overall nine PPV1-positive farms, Cq-values below 30 were present, indicating PPV1 as the possible etiological agent. In two of these cases, PPV1 alone and, in one case, PPV1 in combination with PCV2 were detected. A well-known and broadly accepted association between PPV1 and PCV2 is described in terms of an enhanced outcome of PMWS in cases of double infection [20,47,48]. Concerning SMEDI or other reproductive clinical signs in swine, information is rare. However, a study with the aim of investigating the effect of different parvovirus vaccination schemes in PCV2-infected sows on PMWS in the offspring [6] indicated a protective effect on PCV2-associated reproductive clinical signs and subsequent PMWS in the corresponding offspring. Referring to this, we were able to demonstrate that a PCV2 and PPV1 coinfection was significantly associated with the size, body weight, and mummification of fetuses in our examinations. As the crown-rump length allows to draw conclusions on the age of the corresponding fetus [49,50], this observation points out that the coinfection with both viruses might result in a shorter time to death after infection or a more severe disturbed fetal development. Although numerically different, the viral load estimated by the Cq values in cases of double infection was not significantly affected. This observation indicates an enhanced negative effect concerning the development of a fetus in the case of a double infection with PCV2 and PPV1, but not necessarily due to higher viral loads as observed in growing-to-finish pigs. 

PCV3 was present on 5 out of 18 farms and in a total of 27.8% of all litters. Moreover, 17.5% of the single fetuses were PCV3-positive. Whereas the rate of detection on farm level is comparable to a recent examination on the detection of PCV3 in suckling piglets [31], where no reproductive diseases were evident, our examinations on the occurrence of PCV3 in terms of SMEDI revealed higher litter- or fetus-based levels of detection. The detection rate at farm level is in line with the observation that PCV3 is not as widely spread on the stage of piglet production in Germany as it can be observed for PCV2 [31]. In contrast to this, the higher percentage of positive litters and fetuses might reflect the possible relevance of the PCV3 analog to PCV2 in cases of SMEDI. Considering the assumption of an increased relevance of high viral loads in clinical cases, two farms with a PCV3-Cq-value lower than 30 and a lack of coinfection were present in our examination and therefore suspicious for PCV3-RD with a clinical picture of SMEDI. The association of PCV3 with reproductive disorders was, among others, one of the first described observations concerning the clinical relevance of this virus [51]. Several subsequent publications further strengthen the potential role of PCV3 in terms of reproductive disorders [16,17,27,52], and recently a first proposal on PCV3-associated diseases, including PCV3-RD, was released [19]. Thus, PCV3 should be included in the differential list in cases of SMEDI to provide a complete picture of the infectious status in such cases.

Leptospira spp. were the least frequently detected pathogen in SMEDI-associated fetuses on the farm (11.1%) and reached similar rates of detection as PCV3 concerning single fetuses. The etiological role of Leptospira spp. in SMEDI cases remains uncertain in our study. On the one hand, the Cq-values in positive fetuses were rather high, which points out low bacterial loads in the present cases; on the other hand, the detection of the LipL32-gene, which is found only in pathogenic Leptospira spp. [26], indicates a possible role of Leptospira spp. infections in cases of SMEDI. As mentioned above, we observed a distinct association between the detection of Leptospira spp. and PCV2 in our study, with low-to-medium PCV2-Cq values in such cases. However, this observation points out the possible relevance of PCV2 in cases of Leptospira spp.-associated SMEDI, but to strengthen this hypothesis, a targeted approach is needed as our results have a rather explorative character. Nevertheless, our results support the hypothesis that Leptospira spp. can be involved in the pathogenesis of SMEDI. Interestingly, we recognized a distinct association between the size of the fetuses or a macerated appearance and the detection of Leptospira spp. DNA, either alone, or as a coinfection with PCV2, respectively. This observation is in line with the pathogenesis of maceration in utero, where bacterial infections induce heterolysis followed by the macerated appearance of dead fetuses [53] and points out that leptospiral infection occurs later in the pregnancy. From that point of view, the presence of macerated fetuses in cases of SMEDI or other reproductive disorders indicates bacterial involvement, and leptospiral infection should be included in the laboratory diagnostics in such cases.

## 5. Conclusions

Our examinations reveal that SMEDI has a multi-microbial character, and more than one pathogen can be involved in the pathogenesis at the same time, which indicates the need for a broad laboratory diagnostic approach. Although it has not been described so far, PCV3 should also be considered in cases of SMEDI. Particularly PCV2 + PPV1 and PCV2 + *Leptospira* spp. coinfections showed distinct associations in our examinations. Furthermore, coinfections with PCV2 + PPV1 seem to enhance the negative impact on growth and the time to death of affected fetuses.

## Figures and Tables

**Table 1:** Total number and percentage of available fetuses and the number of enrolled animals under consideration of the phenotype.

Appearance of Fetuses		Total Population	Randomly Selected Study Population
mummified		30.4% (109/358)	30.4% (48/158)
stillborn	macerated	21.5% (77/358)	20.9% (33/158)
	fresh	44.7% (160/358)	41.8% (66/158)
	total	66.2% (237/358)	62.6% (99/158)
weak-born *		3.4% (12/358)	7% (11/158)

* Lung tissue floats on water.

**Table 2:** Detection rates (% and CI) of PPV1, PCV2, PCV3, and *Leptospira* spp. in cases of SMEDI on farm, litter, and fetus level.

Total Detected	PPV1	PCV2	PCV3	*Leptospira* spp.
Farms (n = 18)	50% (27.8–72.2%)	61.1% (38.9–83.3%)	27.8% (11.1–50.0%)	11.1% (0.0–27.8%)
Litters (n = 40)	27.5% (15.0–40.0%)	50.0% (35.0–67.5%)	17.5% (7.5–30.0%)	17.5% (7.5–30.0%)
Fetuses (n = 158)	14.6% (9.5–20.9%)	28.5% (21.5–36.1%)	11.4 (6.3–17.1%)	12.7% (7.6–17.7%)

**Table 3:** Results of the univariate (Chi^2^-test) and the subsequently conducted multivariable analysis, including the odds ratio (OR) and its confidence interval (CI) for each pathogen.

Independent Variable	Dependent Variable	*p*-Value Chi^2^ Test	*p*-Value Binary Logistic Regression	OR	Lower CI	Upper CI
Fetus PCV2 DNA-positive	*Leptospira* spp. DNA-positive	<0.001	<0.001	26.301	4.911	140.870
Fetus stillborn-macerated		0.004	0.003	8.673	2.119	35.488
Gilt		0.023	<0.001	0.006	0.001	0.060
Fetus PPV1 DNA-positive		0.047	<0.001	0.114	0.014	0.490
Sow Lepto.-vacc.		0.045	Not included in the multivariable analysis due to redundant data as no *Leptospira* spp.-vaccinated herds had positive fetuses.			
Fetus *Leptospira* spp. DNA-positive	PCV2 DNA-positive	<0.001	<0.001	9.058	2.772	29.595
PCV3 DNA-positive		0.025	0.008	0.078	0.008	0.742
Sow PCV2-vaccinated		0.001	0.001	0.051	0.007	0.351
Gilt	PPV1 DNA-positive	0.002	-	-	-	-
PCV2 Sow vaccination		0.047	-	-	-	-
Lepto Sow vaccination		0.010	-	-	-	-
Fetus *Leptospira* spp. DNA-positive		0.047	-	-	-	-
PCV2 DNA-positive	PCV3 DNA-positive	-	-	-	-	-
Gilt		-	-	-	-	-

**Table 4:** Qualitative PCR results (% and CI) including coinfections on the farm, litters, and individual fetuses.

(Co) Infections	PPV1	PCV2	PCV3	Lepto. ^1^	PCV2 + PPV1	PCV2 + Lepto. ^1^	PCV3 + Lepto. ^1^	PCV2 + PCV3 + Lepto. ^1^	No Pathogen
Farms (n = 18)	16.7% (0.0– 33.3%)	16.7% (0.0– 33.3%)	16.7% (0.0– 33.3%)	-	33.3% (11.1– 55.6%)	-	-	11.1% (0.0– 27.8%)	5.6% (0.0– 16.7%)
Litters (n = 40)	12.5% (2.5– 22.5%)	22.5% (10.0– 35.0%)	12.5% (2.5– 24.9%)	-	15.0% (5.0– 27.5%)	10.0% (2.5– 20.0%)	2.5% (0.0– 7.5%)	2.5% (0.0– 7.5%)	22.5% (10.0– 37.5%)
Fetuses (n = 158)	8.9% (4.4– 13.3%)	14.6% (9.5– 20.3%)	9.5% (5.1– 14.6%)	2.5% (0.6– 5.1%)	5.7% (2.5– 9.5%)	8.2% (4.4– 12.7%)	1.3% (0.0– 3.2%)	0.6% (0.0– 2.5%)	48.7% (40.5– 55.7%)

^1^ *Leptospira* spp.

**Table 5:** Cq-values with standard deviation for each single pathogen in the case of a mono- or coinfection.

	Mean Cq-Value of Single Pathogens in the Tissue Pools			
(Co)infections	PPV1	PCV2	PCV3	*Leptospira* spp.
PPV1 (n = 14)	27.21 (±1.04)	-	-	-
PCV2 (n = 23)	-	27.41 (±8.28)	-	-
PCV3 (n = 15)	-	-	26.07 (±7.57)	-
*Leptospira* spp. (n = 4)	-	-	-	36.75 (±2.07)
PPV1 + PCV2 (n = 9)	21.96 (±11.23)	32.77 (±1.77)	-	-
PCV2 + *Leptospira* spp. (n = 13)	-	31.21 (±1.98)	-	36.38 (±1.44)
PCV3 + *Leptospira* spp. (n = 2)	-	-	33.69 (±3.49)	33.50 (±0.70)
PCV2 + PCV3 *+ Leptospira* spp. (n = 1)	-	30.09 (±0)	32.43 (±0)	37.00 (±0)

## Data Availability

Data is available from the corresponding author upon reasonable request.

## Supplementary Materials

The following supporting information can be downloaded at: https://www.mdpi.com/article/10.3390/microorganisms11071675/s1, Supplementary File S1: Table of the herd size, vaccination against PPV1, PCV2, and *Leptospira* spp., available litters and fetuses, and examined fetuses; Supplementary File S2: Table of the qualitative PCR results for each farm, including the litters, fetuses, and total farm status; Supplementary File S3: Boxplot of Cq-values for each single pathogen and farm.
