# Supplementary material for: Examination on the Occurrence of Coinfections in Diagnostic Transmittals in Cases of Stillbirth, Mummification, Embryonic Death, and Infertility (SMEDI) Syndrome in Germany
Source: Microorganisms. 2023 Jun 27;11(7):1675. doi: 10.3390/microorganisms11071675 (PMC10383851; doi:10.3390/microorganisms11071675)
Supplement: Supplementary file 1 [file microorganisms-11-01675-s001.zip › Supplemental file S1.pdf]

Table S1: Herd size, vaccination against PPV1, PCV2 and *Leptospira* spp. and available litters and fetuses and examined fetuses.

		Farm number																	
		1	2	3	4	5	6	7	8	9	10	11	12	13	14	15	16	17	18
Herd size Sows (n)		188	280	750	650	650	148	1700	1380	600	2800	180	500	900	1800	83	500	350	240
PCV2 vac.	gilts			X	X	X	X	X		X	X		X	X	X	X	X		
	sows						X			X									
PPV1 vac.	gilts	X	X	X	X	X	X	X	X	X	X	X	X	X	X	X	X	X	X
	sows	X	X	X	X	X	X	X	X	X	X	X	X	X			X	X	X
Leptospira spp. vac.	gilts						X						X						
	sows						X						X						
Quarantine for gilts					X	X		X		X	X	X	X	X			X	X	X
Available litters, n		2	3	2	3	1	4	1	2	1	5	3	2	1	1	1	4	2	2
Available piglets, n		9	18	28	16	4	61	3	18	5	71	40	13	4	12	5	30	8	13
Examined piglets, n		8	12	8	12	4	16	3	8	4	20	12	8	4	4	4	16	7	8
