# Supplementary material for: Examination on the Occurrence of Coinfections in Diagnostic Transmittals in Cases of Stillbirth, Mummification, Embryonic Death, and Infertility (SMEDI) Syndrome in Germany
Source: Microorganisms. 2023 Jun 27;11(7):1675. doi: 10.3390/microorganisms11071675 (PMC10383851; doi:10.3390/microorganisms11071675)
Supplement: Supplementary file 1 [file microorganisms-11-01675-s001.zip › Supplementary file S2.pdf]

Table S2: Qualitative PCR results for each farms including the litters, fetuses and the total farm status.

	PCV2		PPV		PCV3		Leptospira ssp.		
farm	litters	fetuses	litters	fetuses	litters	fetuses	litters	fetuses	total farm status
1	0/1	0/8	0/1	0/8	0/1	0/8	0/1	0/8	-
2	0/4	0/12	0/4	0/12	**3/4**	**9/12**	0/4	0/12	PCV3
3	**1/2**	**1/8**	0/2	0/8	**1/2**	**4/8**	**2/2**	**4/8**	PCV2+PCV3+Lepto
4	**3/3**	**5/12**	0/3	0/12	0/3	0/12	0/3	0/12	PCV2
5	**1/1**	**1/4**	0/1	0/4	0/1	0/4	0/1	0/4	PCV2
6	0/4	0/16	0/4	0/16	**1/4**	**1/15**	0/4	0/16	PCV3
7	**1/1**	**1/3**	**1/1**	**2/3**	0/1	0/3	0/1	0/3	PCV2+PPV
8	**2/2**	**3/8**	**1/2**	**1/8**	0/2	0/8	0/2	0/8	PCV2+PPV
9	0/1	0/4	0/1	0/4	**1/1**	**3/4**	0/1	0/4	PCV3
10	**5/5**	**17/20**	0/5	0/20	**1/5**	**1/20**	**4/5**	**16/20**	PCV2+PCV3+Lepto
11	**1/3**	**8/12**	**1/3**	**3/12**	0/3	0/12	0/3	0/12	PCV2+PPV
12	**2/2**	**5/8**	**2/2**	**8/8**	0/2	0/8	0/2	0/8	PCV2+PPV
13	0/1	0/4	**1/1**	**3/4**	0/1	0/4	0/1	0/4	PPV
14	**1/1**	**1/4**	**1/1**	**2/4**	0/1	0/4	0/1	0/4	PCV2+PPV
15	0/1	0/4	**1/1**	**1/4**	0/1	0/4	0/1	0/4	PPV
16	**1/4**	**2/16**	**2/4**	**2/16**	0/4	0/16	0/4	0/16	PCV2+PPV
17	0/2	0/7	**1/2**	**1/7**	0/2	0/7	0/2	0/7	PPV
18	**1/2**	**1/8**	0/2	0/8	0/2	0/8	0/2	0/8	PCV2
**all**	**19/40**	**45/158**	**11/40**	**23/158**	**7/40**	**18/158**	**6/40**	**20/158**	
