# Supplementary figures and images for: Examination on the Occurrence of Coinfections in Diagnostic Transmittals in Cases of Stillbirth, Mummification, Embryonic Death, and Infertility (SMEDI) Syndrome in Germany
Source: Microorganisms. 2023 Jun 27;11(7):1675. doi: 10.3390/microorganisms11071675 (PMC10383851; doi:10.3390/microorganisms11071675)

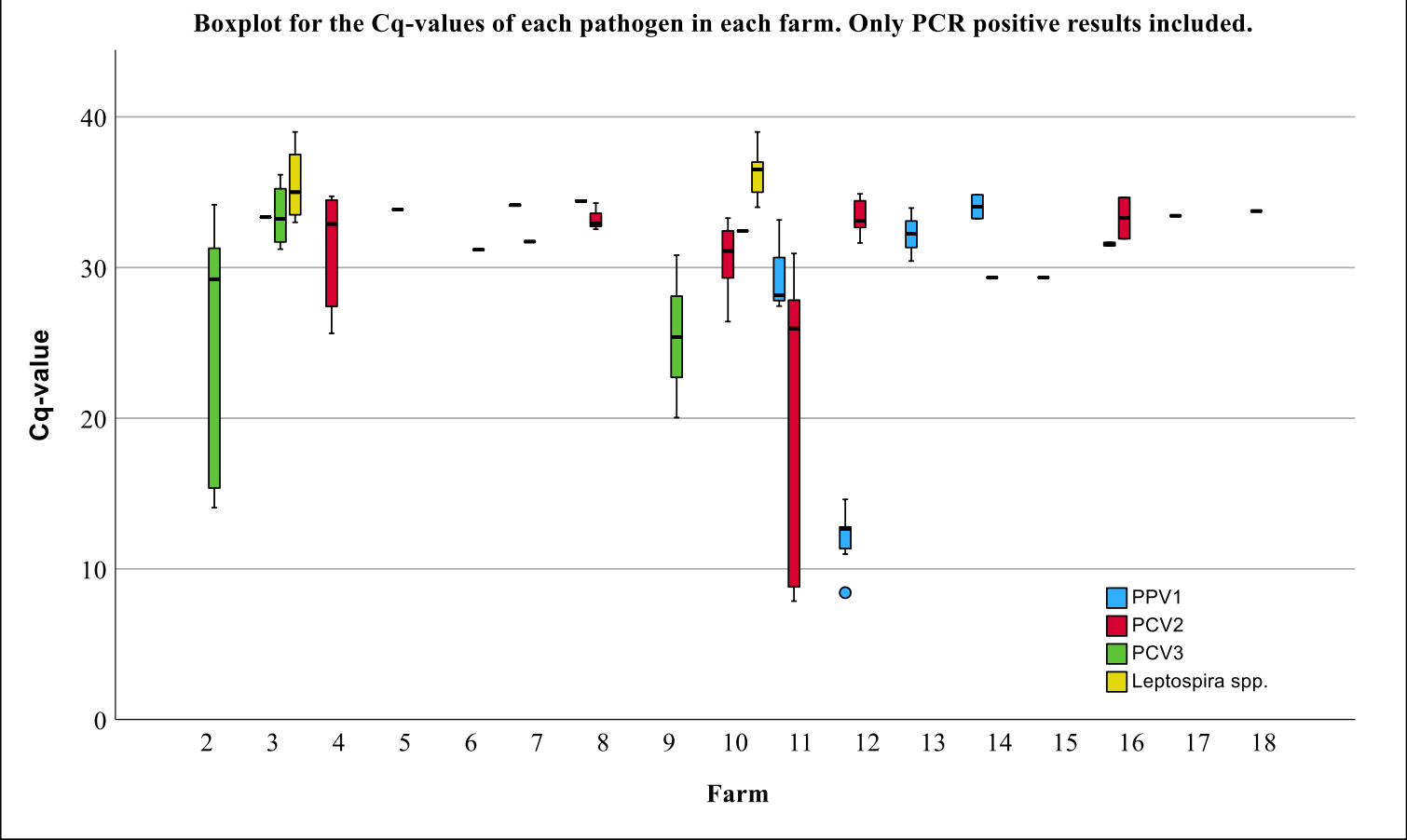

Figure S1: Boxplot of Cq-values for each single pathogen and farm.

Supplement: Supplementary file 1 [file microorganisms-11-01675-s001.zip › Supplementary file S3.pdf]
